# Evaluation of the Colonization of Plants from Five *Quercus* Taxa Native to Greece by *Tuber aestivum* (Ascomycota, Pezizales)

**DOI:** 10.3390/life14070852

**Published:** 2024-07-07

**Authors:** Vassileios Daskalopoulos, Elias Polemis, Irini-Evangelia Kioupidi, Panayiotis Trigas, Georgios I. Zervakis

**Affiliations:** 1 Laboratory of General and Agricultural Microbiology, Agricultural University of Athens, Iera Odos 75, 11855 Athens, Greece; vassilismks@gmail.com (V.D.); eirini.kioup@gmail.com (I.-E.K.); 2 Laboratory of Systematic Botany, Agricultural University of Athens, Iera Odos 75, 11855 Athens, Greece; trigas@aua.gr

**Keywords:** ascospore, ectomycorrhiza, Mediterranean, oak, truffle

## Abstract

Fungi of the genus *Tuber* are famous for their hypogeous ascomata (truffles), many of which possess noteworthy organoleptic properties. *T. aestivum* shows a wide geographic distribution, has many plant symbionts and is well adapted to various climatic conditions. In this study, five *Quercus* taxa native to Greece (i.e., *Q. coccifera*, *Q. ilex*, *Q. ithaburensis* subsp. *macrolepis*, *Q. pubescens* and *Q. trojana* subsp. *trojana*) were inoculated with spore suspensions obtained from a single ascoma of *T. aestivum*. The fungal colonization of oak roots was evaluated at three, seven and 12 months after inoculation; the respective colonization rates for each time period were as follows: low to medium (17–41%) for *Q. pubescens*, *Q. ithaburensis* subsp. *macrolepis* and *Q. trojana* subsp. *trojana*, medium to relatively high (58–80%) for *Q. ithaburensis* subsp. *macrolepis*, *Q. ilex*, *Q. pubescens* and *Q. trojana* subsp. *trojana*, and medium to high (45–87%) for all oak species examined. Positive correlations were assessed between the number of colonized root tips and the total root tips number, but no significant differences were detected between the inoculated plants and the respective control as regards plant growth. The ectomycorrhizae formed by *T. aestivum* with *Q. ithaburensis* subsp. *macrolepis* and *Q. trojana* subsp. *trojana* are described for the first time. The outcome of the study evidences the feasibility of generating the seedlings of various indigenous oak species (covering a large range of diverse habitats) successfully inoculated with autochthonous truffles to be readily used for cultivation purposes.

## 1. Introduction

The genus *Tuber* P. Micheli ex F.H. Wigg. includes fungi forming hypogeous ascomata (truffles), famous for their unique organoleptic properties [1]. *Tuber* species form obligate ectomycorrhizal (ECM) associations with various plants, often with both angiosperms and gymnosperms behaving thus as host generalists [1].

Members of the genus *Tuber* are widely distributed in Europe, Asia and N. America [2], but historically the center of human engagement/occupation with truffles is Europe and particularly the Mediterranean region [3]. This is also the area where most of the gastronomically appreciated truffle species are found, e.g., *Tuber aestivum* Vittad., *T. bituminatum* Berk. & Broome, *T. borchii* Vittad., *T. brumale* Vittad., *T. macrosporum* Vittad., *T. magnatum* Picco, *T. melanosporum* Vittad. *T. mesentericum* Vittad. and *T. suave* Pacioni & M. Leonardii [4,5,6]. Large amounts of truffles are collected every year in Europe; however, a gradual decline in their natural populations is being noted, especially as regards *T. melanosporum* and *T. magnatum* [7,8]. Such decline could be attributed to anthropogenic, sociological or climatic factors related to the changes in land use, overharvesting, global warming, etc. [8,9,10].

The high commercial value of several truffle species and the above-mentioned decline in natural populations led to the development and spread of their cultivation in Europe and elsewhere [3,11,12,13,14,15]. Truffle cultivation is considered a promising agricultural practice with considerable potential for further increase, the most essential factors being the use of high-quality inoculated plants and the appropriate selection of truffle species in conjunction with the suitable edaphoclimatic conditions in the orchard [3,7].

*T. aestivum* is among the most common and highly appreciated truffles in Europe. It presents a relatively wide range of distribution and plant symbionts as well as good adaptation to varying climatic and soil conditions [16,17]; hence, it is more suitable for cultivation in relatively hot and dry environments, in contrast to other more demanding *Tuber* species [18]. The natural distribution of this species extends from North Africa to South Sweden and from Portugal to the Caucasus region, covering a wide range of habitats [16]. *T. aestivum* is associated with many forest tree species, either broadleaves or conifers, such as oak (*Quercus*), beech (*Fagus*), ash (*Tilia*), poplar (*Populus*), birch (*Betula*), fir (*Abies*), spruce (*Picea*) and pine (*Pinus*) [19]. In addition, *T. aestivum* grows in soils of varying composition and structure, from sandy to clayey, with an adequate calcium content [17]. In terms of pH, although many commercial truffle species seem to prefer slightly alkaline soils [20], *T. aestivum* is found in soils ranging from slightly acidic to alkaline in the vicinity of limestone [17]. The species’ fruiting season extends throughout the year, presenting two main peaks during the favorable periods of spring and autumn, depending on the latitude, altitude and microclimate of each region [18,21,22]. The wide range of favorable soils, climatic conditions and host plants, combined with the market value of *T. aestivum* and its long harvest season, makes this species a solid candidate for cultivation [16]. *T. aestivum* has been cultivated in France for more than 40 years, as well as in several other European countries, e.g., Italy, Spain, Sweden, Hungary and Austria [3,16].

Truffle cultivation in Greece is not as developed as in other European countries [3,23]; however, local production exhibits an increasing trend in response to the recent surge in demand. Imported seedlings are mainly inoculated with *T. melanosporum*, which increases the initial investment costs and the risk of introducing an alien species since the “Périgord truffle” is not native to Greece (Daskalopoulos et al.; unpublished data). Hence, the cultivation of a naturally occurring and more adaptive species, such as *T. aestivum*, could be advantageous. Until now, *T. aestivum* has been cultivated in a few orchards only since it usually serves as a “downgraded alternative” in areas where *T. melanosporum* cannot thrive.

The aim of the present study was to investigate and evaluate the colonization of native-to-Greece *Quercus* spp. by using an indigenous strain of *T. aestivum*. Five out of the 14 *Quercus* spp. occurring in Greece [24], namely the evergreen sclerophyllous *Q. coccifera* L. and *Q. ilex* L., the semi-evergreen *Q. ithaburensis* subsp. *macrolepis* (Kotschy) Hedge & Yalt., and the deciduous *Q. pubescens* Willd. and *Q. trojana* Webb subsp. *trojana*, were inoculated with spore suspensions obtained from a single ascoma of *T. aestivum*. The main aim of the study was to verify to what extent and how rapidly a symbiotic relationship between the two parts could be established. In addition, seedling growth was measured to determine the possible effect of colonization on plant development. The basic morphoanatomic features of the five ECM associations were noted and compared to the pertinent literature. Two of them (i.e., those formed by *T. aestivum* with *Q. ithaburensis* subsp. *macrolepis* and *Q. trojana* subsp. *trojana*) are described for the first time.

## 2. Materials and Methods

### 2.1. Plant Material

**Figure 1:**
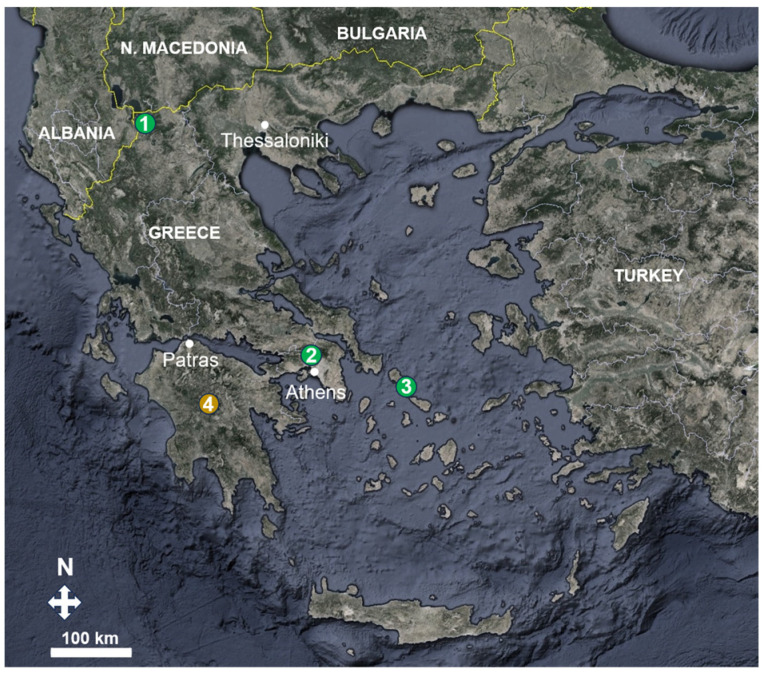
Map of Greece depicting collection sites of oak acorns (green dots) and of *Tuber aestivum* ascoma (yellow dot); (1) Prespes, (2) Athens, (3) Andros Isl., (4) Arcadia.

Acorns from five *Quercus* species were collected from three areas in Greece, i.e., *Q. coccifera* (campus of the Agricultural University of Athens, Central Greece), *Q. ilex* (Andros Isl., Aegean Sea), *Q. ithaburensis* subsp. *macrolepis* (Andros Isl., Aegean Sea), *Q. pubescens* (Andros Isl., Aegean Sea) and *Q. trojana* subsp. *trojana* (Prespes, NW Greece) (Figure 1). The seeds were disinfected in a 1% (*v*/*v*) bleach solution for 1 h [25], and then were placed in sterile sand at 4 °C (wet stratification) from the date of collection to the date of sowing.

### 2.2. Fungal Inoculum

For the preparation of the inoculum, a fully mature ascoma of *T. aestivum* was collected from a forest dominated by *Abies cephalonica* Loudon in the Arcadia region (Peloponnese, South Greece) (Figure 1). The specimen was morphoanatomically identified according to Leonardi et al. [5], and the result was verified via ITS rDNA sequencing (GenBank accession no. PP725740) and performed as previously described [26]. The *T. aestivum* ascoma was placed into a freezer until inoculation; then, 25 g of gleba was placed in a homogenizer (previously disinfected with ethanol) with 500 mL of sterile water, and stirred until a homogeneous mixture was obtained. Sterile water was added to a final volume of 2.5 L, and the resulting suspension was measured to contain 6 × 10^4^ ascospores mL^−1^.

### 2.3. Preparation and Inoculation of Oak Seedlings

The sowing of the acorns was performed in trays with sterile sand which were then placed in growth chambers (Conviron GEN1000, Conviron, Winnipeg, MB, Canada). The environmental conditions within the chambers were initially set as follows: 18 h of light, temperature 22 °C, RH 85%, 6 h of darkness, temperature 20 °C and RH 70%. At the stage of the first two to four real leaves, the young seedlings were transferred to individual pots (volume of 750 mL), after suitably pruning the long central root that they had developed, leaving a part measuring approx. 10 cm. The growth substrate was a sterilized mixture (1:1:1 *v*/*v*) of equilibrated peat, vermiculite and natural calcareous soil (CL/SiCL, soil organic matter ~1.5%, CaCO_3_: 28.7%, pH: 8.0). The young seedlings were allowed to grow for 1 to 1.5 months. Then, 20 seedlings from each one of the five oak species were inoculated with the ascospore suspension; each seedling received 25 mL of the suspension (i.e., approx. 1.5 × 10^6^ spores) by surface application. Five plants per species were not inoculated and served as the control.

### 2.4. Growth of Oak Plants and Description of ECM associations

After the inoculation procedure, the seedlings were transferred to a more spacious custom-made growth chamber, in which they remained throughout the experiment, under the following conditions: 18 h of light with an average temperature of 26 °C and RH of 70% and 6 h of darkness with an average temperature of 17 °C and RH of 70%. Throughout the experiment, the seedlings were irrigated two to three times per week depending on their needs.

Five seedlings per oak species were examined to evaluate the colonization at three, seven and twelve months after inoculation, and to measure plant growth (stem diameter at the substrate level and height); five non-inoculated plants per species served as the control for the latter experiment. The colonization was estimated by counting the colonized root tips vs. the total number of root tips (expressed as % values) for each plant and at each evaluation period, according to the general guidelines of Alpuente et al. [27] and Donnini et al. [28]. As regards the non-inoculated plants (control), the root system was examined at the end of the experiment (12-month period) only. The seedlings’ root system was rinsed with tap water to remove the soil, and then the plants were placed in jars with water prior to their examination.

Oak roots were examined macroscopically and with the aid of a stereoscope (ΝΙΚOΝ SMZ18, Nikon Corporation, Tokyo, Japan) to evaluate the growth and colonization by the fungus. Photographs of the root tips were obtained with the aid of the OCULAR Software (Teledyne Photometrics, Tucson, AZ, USA). The anatomical features were studied using a microscope (Olympus BX53F2, Olympus Corporation, Tokyo, Japan), while the respective images were obtained by using a digital camera (Olympus DP74, Olympus Corporation, Tokyo, Japan) and processed with cellSens Entry software (Olympus Life Science, Waltham, MA, USA). The examination of the morpho-anatomical characters of ectomycorrhizal or non-ectomycorrhizal root tips followed Agerer [29] and Agerer and Rambold [30]. The description of the morpho-anatomical features of *T. aestivum* ectomycorrhizae followed Zambonelli et al. [31].

### 2.5. Statistical Analysis

Pearson’s correlation test, Duncan *t*-test and one-way ANOVA at a 95% significance level were performed on the results obtained by using MS Excel (MS Office 2019, Microsoft Corporation, Redmond, WA, USA), GraphpadPrism v.5 (Graphstats Technologies, Bangalore, India) and IBM SPSS Statistics v.20 (IBM Corporation, Armonk, NY, USA), respectively.

## 3. Results

### 3.1. Colonization of Oak Roots by T. aestivum

**Figure 2:**
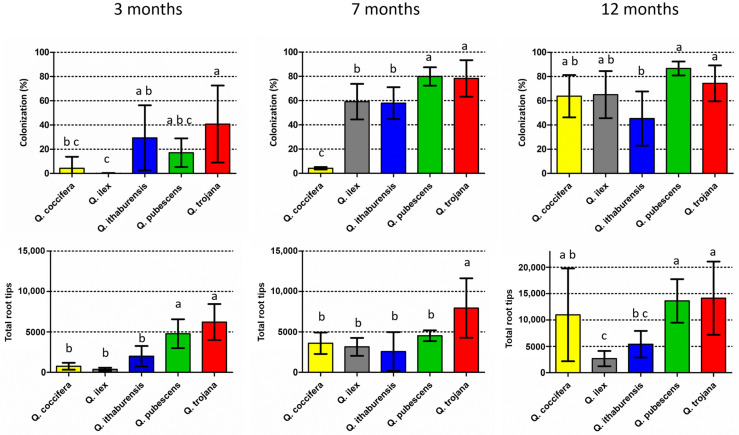
Mean colonization rates (number of colonized root tips vs. number of total root tips, %) (above), and mean number of total root tips (below) per evaluation period (three, seven and twelve months). Vertical bars on the columns represent the standard deviation (SD), while absence of common letters indicates significant differences (*p* < 0.05) in comparisons among plant species.

Colonized root tips by *T. aestivum* were found at various rates in all *Quercus* species examined three months after inoculation (Figure 2; Supplementary Material, Table S1). The lowest mean colonization rate was noted in the evergreen *Quercus* spp. (i.e., *Q. ilex* 0.1% and *Q. coccifera* 4.3%), where colonized root tips were detected in only one of the five individual seedlings examined per plant species. The highest colonization (i.e., *Q. trojana* subsp. *trojana* 41%, *Q. ithaburensis* subsp. *macrolepis* 29% and *Q. pubescens* 17%) was observed in the deciduous oaks which possessed colonized root tips in all five seedlings of each plant species. When the colonization rates were compared, significant differences were noted for *Q. trojana* subsp. *trojana* vs. *Q. coccifera* and *Q. ilex*, and for *Q. ithaburensis* subsp. *macrolepis* vs. *Q. ilex*. Similarly, *Q. pubescens* and *Q. trojana* subsp. *trojana* showed significantly higher values regarding the number of total root tips in comparison to the other oak species. The highest colonization per individual seedling was observed in *Q. ithaburensis* subsp. *macrolepis* (73%) and *Q. trojana* subsp. *trojana* (71%), while the highest number of total root tips was detected in *Q. trojana* subsp. *trojana*. Nevertheless, individual seedlings with very low colonization (<6%) were observed in all deciduous species as well.

Seven months after inoculation, colonized root tips were found in all the individual seedlings examined, and colonization increased considerably in all the species except for *Q. coccifera*, while the total number of root tips increased in all the species except for *Q. pubescens* (Figure 2; Supplementary Material, Table S1). *Q. pubescens* and *Q. trojana* subsp. *trojana* demonstrated significantly higher colonization when compared to the other species, whereas *Q. coccifera* exhibited the lowest colonization. In addition, *Q. trojana* subsp. *trojana* showed significantly higher numbers of total root tips in comparison to all the other species (Figure 2). When the results of the 3-month and 7-month periods were compared, *Q. ilex*, *Q. pubescens* and *Q. trojana* subsp. *trojana* showed significant increases in their respective colonization rates, but only *Q. ilex* presented a significant increase in the total number of root tips as well (Supplementary Material, Figure S1). Interestingly, the total number of root tips in *Q. coccifera* increased fivefold from the 3-month to the 7-month period although the colonization remained low (4%) (Supplementary Material, Table S1). The highest colonization was observed for *Q. pubescens* (ca. 80%), which corresponded to a considerable increase over the 3-month period (Supplementary Material, Table S1 and Figure S1). In the case of *Q. ilex*, both colonization and total root tip number increased significantly, while *Q. trojana* subsp. *trojana* showed high levels of root colonization (ca. 78%) and the highest number of total root tips among the oak species examined.

Lastly, as concerns the 12-month period, all five *Quercus* species exhibited medium to high colonization rates (>45%) (Figure 2). A significant increase in relation to the 7-month period was observed in *Q. coccifera* only, for both colonization and the total number of root tips; the other four oak species showed similar levels of colonization as in the 7-month period, but with a considerable rise in the total number of root tips, with the only exception of *Q. ilex* (Figure 2; Supplementary Material, Table S1 and Figure S1). The non-inoculated (control) seedlings were examined at the 12-month period, and exhibited no signs of contamination.

**Figure 3:**
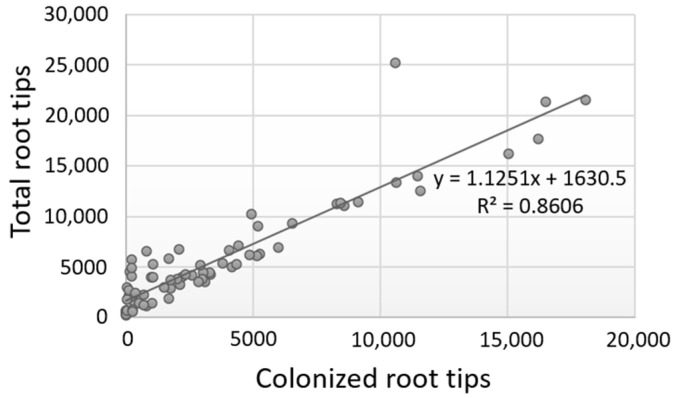
Correlation of the number of colonized root tips vs. the total number of root tips for all data obtained from the three evaluation periods and the five *Quercus* spp. examined.

Further tests evidenced that the number of colonized root tips vs. the total number of root tips was highly correlated for each evaluation period or for each plant species examined, and for all values combined (Pearson correlation values ≥ 0.80) (Figure 3; Supplementary Material, Table S2). These results indicate that the successful ECM colonization of plants heavily depends on the good development of the root tips; hence, higher colonization rates are a consequence of a higher number of total root tips as exemplified in the case of deciduous oaks, whereas reduced colonization is due to the production of fewer root tips as observed in the case of evergreen oaks. However, the exceptions noticed in individual trees suggest that other factors (e.g., intrinsic) might also influence the colonization process.

### 3.2. Plant Growth and Root Structure

**Figure 4:**
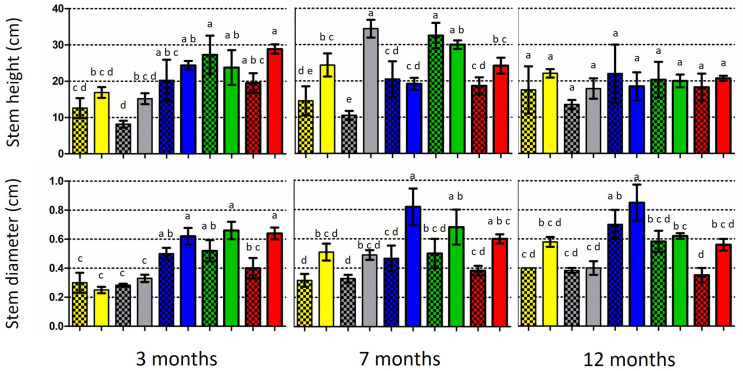
Growth of five *Quercus* species at three time periods: mean stem height (above) and mean stem diameter at substrate level (below) of inoculated (light columns) and non-inoculated (control; dark/shaded columns) plants. Vertical bars on the columns represent the standard deviation (SD) of means, while significant differences (*p* < 0.05) are indicated by absence of common letters on the columns (comparisons were made among inoculated plants and the controls for each time period).

At the end of the 3-month period, all the inoculated oak species showed a higher stem height and stem diameter than the control seedlings, with the exceptions of *Q. pubescens* and *Q. coccifera*, respectively (Figure 4; Supplementary Table S3). However, only in the case of the stem diameter in *Q. trojana* subsp. *trojana* was a significantly larger value detected in the comparisons between the inoculated and control plants. At the end of the 7-month period, the inoculated seedlings of two out of five *Quercus* spp. (i.e., *Q. coccifera* and *Q. ilex*) exhibited significantly higher stems when compared to the respective controls. Similarly, the inoculated seedlings of all the *Quercus* species possessed larger stems, but only *Q. ithaburensis* subsp. *macrolepis* demonstrated significant differences in respect to the control. Last, at the end of the 12-month period, the inoculated seedlings of all the *Quercus* species exhibited a higher stem height and larger stem diameter in comparison to the control, but no significant differences were noted (Figure 4; Supplementary Material, Table S3).

In addition, the colonization rates and respective plant growth were not correlated (when compared for each evaluation period and *Quercus* species examined), with the only exception of the stem height and diameter in *Q. coccifera* (Pearson correlation values 0.73 and 0.80, respectively). A threshold of growth decrease or stagnancy was observed between the 7- and 12-month periods in all the plants (Figure 4), indicating the gradual prevalence of adverse effects, most possibly due to the limitations imposed by the size of the pot and/or the conditions in the growth chamber.

**Figure 5:**
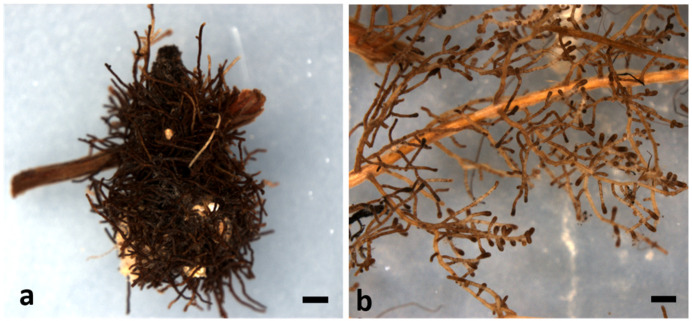
Indicative macro-morphoanatomical features of root systems of the oak species studied, and their ectomycorrhizae: (**a**) dense clusters of root tips on the root system of *Q. coccifera*, and (**b**) sparse colonized root tips on the root system of *Q. trojana* subsp. *trojana*. Scale bars correspond to 1 mm.

As regards the root system of the oak seedlings, it was found to be healthy in all the cases, with sufficient biomass developed from the secondary roots. The root system architecture was relatively well balanced and representative of this particular type of ECM symbiosis (Supplementary Table S4), except for the malformations/abnormalities present either at the upper part (i.e., oblique, woody twists observed already from the seed tray stage) or at the lower bottom part (i.e., circular twists due to the shape of the individual pots used) of the plants. In general, the oak seedlings of the species under study presented two growth patterns: (a) the formation of dense clusters of root tips (Figure 5a), and/or (b) the formation of a uniformly arranged root system with abundant solitary and dense root tips (Figure 5b). All the oak species formed clusters of dense root tips, but in the evergreen and semi-evergreen species, this pattern was more prominent. *Q. coccifera* formed the largest and more abundant clusters showing a pattern of a dominant central root with clusters being mainly present in the upper part of the root system, while the lower parts were relatively bare, exhibiting scattered root tips only. In contrast, the deciduous oak species—although they also possessed a dominant central root—developed many lateral roots with abundant solitary root tips. The weakest clustering was noticed in *Q. trojana* subsp. *trojana*, which showed a pattern of a (uniformly) very dense root system with abundant solitary root tips. In general, the root tip clusters were mainly observed in the upper part of the root system of the inoculated plants, but they also appeared—albeit to a lesser extent and intensity—on the control (non-inoculated) seedlings.

### 3.3. Description of the Ectomycorrhizae Formed by Quercus spp. and T. aestivum

**Figure 6:**
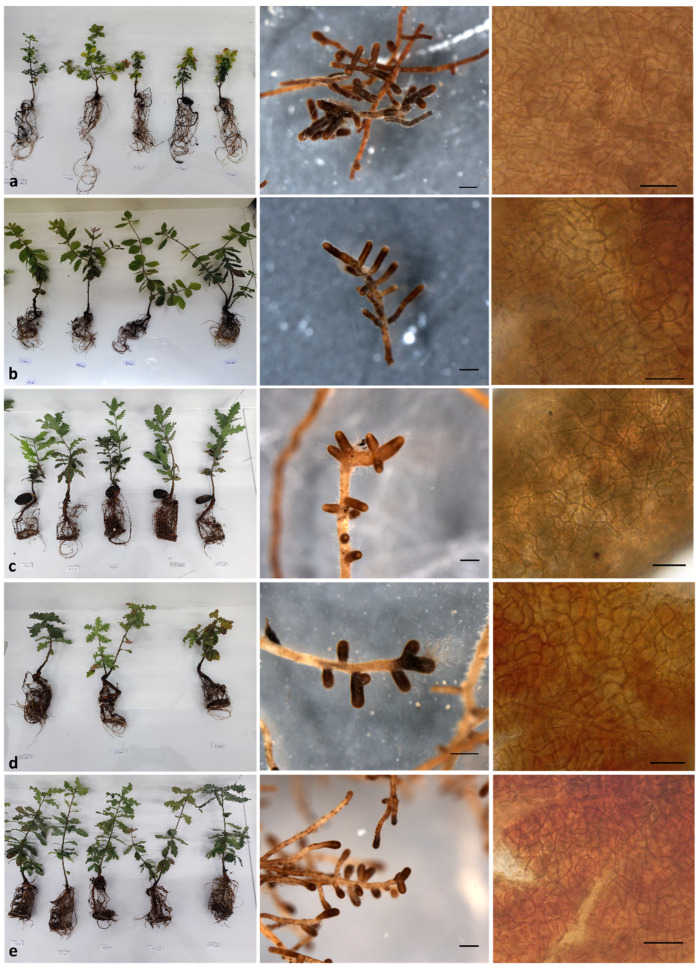
Indicative appearance of the bare roots (left column), the morphology of ectomycorrhizae (central column) and the anatomy of their outer mantle (right column) for (**a**) *Q. coccifera*, (**b**) *Q. ilex*, (**c**) *Q. ithaburensis* subsp. *macrolepis*, (**d**) *Q. pubescens*, and (**e**) *Q. trojana* subsp. *trojana*. Scale bars of the stereoscopic photos correspond to 0.5 mm; scale bars of microscopic photos correspond to 20 μm.

All ECM associations formed by *Quercus* spp. inoculated with *T. aestivum* showed similar morphoanatomic features which can be summarized as follows: a mycorrhizal system of the contact exploration type, either solitary with no ramification, or with a monopodial pinnate to monopodial pyramidal structure; unramified root tips which were straight, cylindrical or inflated, club shaped, with brownish, brown to dark brown colors, depending on their maturity and age; a shiny surface, loosely to densely wooly, or densely long spiny, due to cystidioid emanating hyphae appearing predominantly apically (occasionally along the entire root tip surface); a mantle structure which is plectenchymatous when immature, with a ring-like arrangement (type A), net-like arrangement with repeated and squarrose branches (type E) and/or with a net of coarse and irregularly shaped hyphae (type H), then gradually becoming pseudoparenchymatous, composed of epidermoid (type M) to angular cells (type L), and pseudoparenchymatous with angular cells (type L) when mature (Figure 6); a mature outer mantle layer pseudoparenchymatous with angular cells (type L), sometimes with dense or scattered projecting cystioid hyphae; an outer mantle cell with a triangular, quadrilateral or polygonal shape, with an average density of approximately 5–9 per 20 × 20 μm^2^; emanating bristle-like and wavy hyphae (type A), membranaceously yellowish, without contents, of an equal width and smooth surface. A more detailed presentation of all the ectomycorrhizal morphoanatomic features is provided in the Supplementary Material, Table S4.

## 4. Discussion

Our results demonstrate that *T. aestivum* formed symbiotic relationships with all the inoculated *Quercus* spp., and that the onset of symbiosis could occur quite early. In general, the colonization process progressed at various rates/intensities among the *Quercus* species examined; however, a specific pattern was detected for the evergreen, semi-evergreen and deciduous oaks. Initially, the two sclerophyllous evergreen species (*Q. coccifera* and *Q. ilex*) showed poor colonization and lower numbers of total root tips when compared to the semi-evergreen and deciduous oaks. The latter exhibited high colonization rates for such a short time after inoculation, and a relatively high number of total root tips. Similarly, in the 7-month and 12-month periods, high colonization and root system growth were consistently observed for the two deciduous oaks, while the two evergreen oaks and the semi-evergreen *Q. ithaburensis* subsp. *macrolepis* exhibited lower values. Ultimately, all the oak species reached a threshold/plateau regarding the root colonization within the 12-month period. However, it should be noted that this threshold seemed to be a consequence of the formation of abundant circular and degraded roots (mostly with uncolonized root tips) growing under rather anoxic conditions at the bottom of the pots. This was more evident in the fast-growing deciduous oaks developing abundant roots (and fewer root tips clusters) at the lower part of the pot, for which the threshold in the colonization rate appeared earlier, i.e., in the 7-month period. In contrast, *Q. coccifera*, growing slower than the other species, seemed to be less affected and exhibited an increase in colonization from seven to twelve months.

The pertinent literature reported that a period of about two to three months is required for the initiation of the colonization process in various truffles and host trees. Specifically, Giomaro et al. [32], who inoculated *Tilia platyphyllos* Scop. seedlings with five *Tuber borchii* pure cultures and examined weekly their root system, reported that the first ectomycorrhizas were observed at three months, while at four months, the colonization rates ranged from 51% to 82%. The in vitro co-cultivation of *Cistus incanus* L. and *T. melanosporum* resulted in the establishment of a mycorrhizal association in three months, whereas complete, mature root tips were observed at five months after inoculation [33]. Ori et al. [25] inoculated *Quercus robur* L. seedlings with *T. aestivum* ascospores (derived from ascomata previously fed to slugs and mice), and recorded colonization rates of ca. 10% in three months. In general, the literature reports concur that the colonization rates rarely exceed 15% in the first three months, but eventually *Tuber* species colonize the plant’s root system by exhibiting high values (i.e., from 42 to 81%) at 12 months after inoculation [34,35,36].

Only a few studies exist regarding the colonization of forest trees by *Tuber* spp. and the repercussions on plant development. Some of them reported a positive effect of *Tuber* symbiosis on plant growth: *Q. mongolica* Fisch. ex Ledeb. Seedlings’ colonization by *T. melanosporum* contributed to the regulation of the carbon economy and (by affecting the rhizosphere’s bacterial communities) promoted plant growth and nutrient cycling [37], while *Castanopsis rockii* A.Camus seedlings’ colonization by *T. indicum* Cooke & Massee and *T. lijiangense* L. Fan & J.Z. Ca significantly increased the leaf photosynthetic rate and stimulated the plant growth [38]. In contrast, the investigation of the effect of the co-inoculation of *Q. ilex* seedlings with bacteria along with *T. melanosporum* resulted in similar values of plant (stem) growth for inoculated and non-inoculated plants after six months, while the stem growth either increased or decreased in the presence of bacteria [39]. Ιnterestingly, the stem growth of *Q. ilex* plants decreased as their colonization by *T. melanosporum* increased. Nevertheless, the co-presence of ECM and bacteria positively influenced the root system’s growth and architecture. Alvarez-Lafuente et al. [40] who inoculated *Castanea sativa* Mill. seedlings with *T. aestivum*, reported that after two years, the colonized plants exhibited normal growth with an occasionally larger (albeit not significantly) stem height and diameter than plants inoculated with other *Tuber* spp. In another study, *Quercus robur* and hazelnut seedlings exhibited a significant loss of vitality five months after their inoculation with *T. aestivum* [41]. To the best of our knowledge, this is the first time that the abovementioned plant growth features have been examined for the five *Quercus* species inoculated with *T. aestivum*.

Although the symbiotic relationships between *T. aestivum* and *Q. ilex*, *Q. coccifera* and *Q. pubescens* have already been confirmed and described [42,43], no such reports exist for *Q. trojana* subsp. *trojana* and *Q. ithaburensis* subsp. *macrolepis* [19]. Therefore, on the basis of the available literature data [30,43], it is the first time that the ECM associations formed between *T. aestivum* and *Q. ithaburensis* subsp. *macrolepis* and *Q. trojana* subsp. *trojana* have been recorded. This finding is especially important for habitats dominated by the former plant species, which in general are more arid (e.g., the Mediterranean islands), and they were previously considered not to be favorable for *T. aestivum* harvesting. The respective descriptions reveal that the basic morphoanatomic features of these ECM associations (produced ex situ) exhibited minor differences when compared with those of *T. aestivum* + *Quercus* spp. ECM associations formed in situ [30,31], such as the color of unramified ends (no yellowish brown tints in our specimens), the size of some morphoanatomic characteristics (i.e., unramified ends length and outer mantle cell density) and the plectenchymatous structure of immature mantle at the initial stages of colonization.

The strong positive correlation between the number of total root tips and the colonization rates for all the combinations examined (per each evaluation period, for each *Quercus* species studied and for all values combined) is of high interest. Since root tips (third-order root system formed on secondary roots) constitute the interface where the plant and fungus interact, the results of our research demonstrate the high importance of the development of the lateral, branching root system in the establishment of symbiosis.

The speed and effectiveness of the seedlings’ colonization, absence of contaminations and good plant health are important prerequisites for the success of truffle cultivation. The main ecological advantages which the present work demonstrated are that *T. aestivum* is an appropriate symbiont for all the five *Quercus* species examined, and that colonization was achieved quite fast, reaching a plateau 12 months after inoculation; these advantages have significant economic repercussions in the commercialization of this process. Moreover, since truffle orchards in Greece (and elsewhere) are established by using imported seedlings (i.e., allochthonous biological material—both the plant and fungus—originating from nurseries abroad), our results indicate that local (autochthonous) material could be exploited to reduce the risks of introducing foreign genetic resources into the domestic biodiversity, and to produce inoculated seedlings well adapted to the local conditions.

## 5. Conclusions

*Tuber aestivum* formed symbiotic relationships ex situ with all the five *Quercus* species (no pertinent previous reports exist from natural habitats with *Q. ithaburensis* subsp. *macrolepis* or with *Q. trojana* subsp. *trojana*). The onset of symbiosis can occur quite early. At the 3-month period, trees exhibiting colonization higher than 20% were noted in four out of the five oak species examined. Colonization appears to be positively correlated with the total number of roots in all the oak species. However, exceptions were also noted, suggesting that there are other factors affecting this process. The measurement of the effect of the symbiosis on plant growth did not provide a clear outcome since the stem height was not correlated with the colonization rates, while the stem diameter showed a positive correlation only in the case of *Q. coccifera* seedlings. Significant differences between the inoculated and control seedlings were found only for the stem height at seven months for *Q. coccifera* and *Q. ilex*, and for the stem diameter at three and seven months for *Q.trojana* subsp. *trojana* and *Q. ithaburensis* subsp. *macrolepis*, respectively. To the best of our knowledge, this is the first study assessing plant growth for these particular five oak species when inoculated with *T. aestivum*. Similarly, the ECM associations that developed between *T. aestivum* and *Q. trojana* subsp. *trojana* or *Q. ithaburensis* subsp. *macrolepis* are reported and described for the first time. The basic morphoanatomic features of ECM morphotypes do not appear to differ from the general features exhibited by *T. aestivum* and *Quercus* species under natural conditions. Only the plectenchymatous structure of the immature mantle (at the initial stages of colonization) is slightly different from the typical *T. aestivum* + *Quercus* spp. ECM morphology.

## Data Availability

The data generated in this study are presented in the paper and in the accompanying Supplementary Material.

## Supplementary Materials

The following supporting information can be downloaded at: https://www.mdpi.com/article/10.3390/life14070852/s1, Table S1: Colonization rates (number of colonized root tips vs. number of total root tips, %) and total number of root tips for five *Quercus* species inoculated with *Tuber aestivum* as assessed at three time periods (three, seven and twelve months after inoculation). Values are provided for each replicate (n = 5) per treatment as well as for their means and respective standard deviation (SD). Absence of common superscript letters indicates significant differences (*p* < 0.05) in comparisons among plant species per each time period. Table S2: Pearson correlation coefficient values calculated for the number of colonized rot tips vs. the total number of root tips per evaluation period and plant species (each one and all). Table S3: Stem diameter (at substrate level) and height of five *Quercus* species inoculated (or not, control) with *Tuber aestivum* as assessed at three time periods (three, seven and twelve months after inoculation). Values correspond to means (n = 5) and their standard deviation (SD). Table S4: Detailed morphoanatomic features of the ectomycorrhizae (ECM) studied vs. the general features of *T. aestivum* + *Quercus* spp. ECM associations [31] according to Agerer and Rambold [30]. Figure S1: (a) Mean colonization rates (number of colonized root tips vs. number of total root tips, %) and (b) mean number of total root tips for each *Quercus* species examined. Vertical bars on the columns represent the standard deviation (SD), while absence of common letters indicates significant differences (*p* < 0.05) in comparisons among the evaluation periods (3 m, 7 m, and 12 m: 3-month, 7-month and 12-month, respectively).
