# Supplementary material for: Evaluation of the Colonization of Plants from Five Quercus Taxa Native to Greece by Tuber aestivum (Ascomycota, Pezizales)
Source: Life (Basel). 2024 Jul 7;14(7):852. doi: 10.3390/life14070852 (PMC11277910; doi:10.3390/life14070852)
Supplement: Supplementary file 1 [file life-14-00852-s001.zip › life-3039320-supplementary.pdf]

Supplementary Material, Table S1: Colonization rates (number of colonized root tips vs. number of total root tips, %) and total number of root tips for five *Quercus* species inoculated with *Tuber aestivum* as assessed at three time periods (three, seven and 12 months after inoculation). Values are provided for each replicate (n=5) per treatment as well as for their means and respective standard deviation (SD). Absence of common superscript letters indicates significant differences ($p < 0.05$) in comparisons among plant species per each time period.

	Q. coccifera	***Q. ilex***	***Q. ithaburnesis* subsp. *macrolepis***	***Q. pubescens***	***Q. trojana* subsp. *trojana***
**3-month period**					
**No 1**	0% (0/527)	0% (0/251)	4.37% (30/686)	5.95% (126/2118)	70.63% (3800/5380)
**No 2**	0% (0/569)	0% (0/200)	10.00% (200/2000)	25.00% (1000/4000)	12.21% (800/6550)
**No 3**	0% (0/497)	0.57% (3/525)	72.73% (800/1100)	20.09% (1060/5275)	62.36% (4440/7120)
**No 4**	0% (0/693)	0% (0/638)	27.04% (1070/3957)	3.85% (220/5712)	57.78% (5200/9000)
**No 5**	21.30% (322/1512)	0% (0/289)	32.73% (720/2200)	30.81% (2080/6750)	1.00% (30/3000)
**Mean colonization rate (%)**	**4.26 ± 9.53 ^{b,c}**	**0.11± 0.25 ^{c}**	**29.37 ± 26.91 ^{a,b}**	**17.14 ± 11.82 ^{a,b,c}**	**40.80 ± 31.80 ^{a}**
**Mean number of total root tips**	**759.60 ± 427.18 ^{b}**	**380.60 ± 190.33 ^{b}**	**1988.60 ± 1265.48 ^{b}**	**4771.00 ± 1781.07 ^{a}**	**6210.00 ± 220.41 ^{a}**
**7-month period**					
**No 6**	3.40% (155/4550)	38.60% (537/1391)	61.36% (4050/6600)	74.60% (3305/4430)	92.43% (11600/12550)
**No 7**	3.22% (57/1770)	55.94% (2210/3950)	73.21% (1025/1400)	88.06% (3100/3520)	83.33% (5250/6300)
**No 8**	4.03% (109/2700)	57.13% (1697/2970)	61.37% (1780/2900)	83.80% (4190/5000)	78.18% (8600/11000)
**No 9**	5.70% (230/4035)	65.22% (2110/3235)	56.32% (690/1225)	83.36% (4360/5230)	52.97% (2005/3785)
**No 10**	4.77% (235/4920)	78.76% (3320/4215)	37.24% (270/725)	69.50% (3055/4400)	84.59% (5160/6100)
**Mean colonization rate (%)**	**4.22 ± 1.02 ^{c}**	**59.13 ± 14.65 ^{b}**	**57.90 ± 13.11 ^{b}**	**79.86 ± 7.58 ^{a}**	**78.30 ± 15.05 ^{a}**
**Mean number of total root tips**	**3595.00 ± 1322.20 ^{b}**	**3152.20 ± 1107.69 ^{b}**	**2570.00 ± 2394.36 ^{b}**	**4516.00 ± 662.74 ^{b}**	**7947.00 ± 3672.69 ^{a}**
**12-month period**					
**No 11**	74.19% (8310/11200)	89.47% (1700/1900)	15.30% (375/2450)	93.18% (15050/16150)	83.89% (18080/21550)
**No 12**	42.06% (10580/25150)	62.89% (2610/4150)	70.43% (6550/9300)	81.92% (11470/1400)	81.08% (16500/21350)

**No 13**	74.67% (8460/11330)	51.09% (1497/2930)	29.13% (1690/5800)	91.52% - (16200/17700)	80.26% (9150/11400)
**No 14**	47.72% (1750/3667)	79.47% (3020/3800)	56.34% (2930/5200)	86.95% (6000/6900)	48.14% (4921/10221)
**No 15**	80.28% (2850/3550)	42.39% (251/592)	55.17% (2345/4250)	80.07% (10650/13300)	78.70% (4880/6200)
**Mean colonization rate (%)**	**63.78 ± 17.53 [a,b]**	**65.06± 19.48 [a,b]**	**45.27 ± 22.43 [b]**	**86.73 ± 5.75 [a]**	**74.41 ± 14.81 [a]**
**Mean number of total root tips**	**10979.40 ± 8798.36 [a,b]**	**2674.4 ± 1453.08 [c]**	**5400.0 ± 2521.66 [b,c]**	**13610.0 ± 4136.79 [a]**	**14144.2 ± 6942.66 [a]**

Supplementary Material, Table S2: Pearson correlation coefficient values calculated for the number of colonized rot tips vs the total number of root tips per evaluation period and plant species (each one and all).

All species per evaluation period	Correlation coefficient	All evaluation periods per plant species	Correlation coefficient	Total (all species, all evaluation periods)
3-month	0.800	*Q. coccifera*	0.907	
7-month	0.907	*Q. ilex*	0.967	
12-month	0.920	*Q. ithaburnesis* subsp. *macrolepis*	0.920	
		Q. pubescens	0.956	
		Q. trojana subsp. *trojana*	0.959	
				0.928

Supplementary Material, Table S3: Stem diameter (at substrate level) and height of five *Quercus* species inoculated (or not, control) with *Tuber aestivum* as assessed at three time periods (three, seven and 12 months after inoculation). Values correspond to means (n=5) and their standard deviation (SD).

	**3-month period**		**7-month period**		**12-month period**	
	**Control**	**Inoculated seedlings**	**Control**	**Inoculated seedlings**	**Control**	**Inoculated seedlings**
Q. coccifera						
Stem height (cm)	12.60 ± 6.14	16.90 ± 3.29	14.50 ± 6.95	24.40 ± 7.09	17.50 ± 9.19	22.10 ± 2.61
Stem diameter (cm)	0.30 ± 0.15	0.25 ± 0.05	0.32 ± 0.08	0.51 ± 0.13	0.40 ± 0.00	0.58 ± 0.08
Q. ilex						
Stem height (cm)	8.17 ± 2.29	15.20 ± 3.40	10.50 ± 3.28	34.40 ± 5.50	13.5 ± 2.18	17.90 ± 6.27
Stem diameter (cm)	0.28 ± 0.03	0.33 ± 0.06	0.33 ± 0.07	0.49 ± 0.07	0.38 ± 0.03	0.40 ± 0.11
Q. ithaburnesis* subsp. *macrolepis						
Stem height (cm)	20.25 ± 11.40	24.40 ± 2.63	20.50 ± 8.53	19.20 ± 3.70	22.00 ± 11.31	18.50 ± 8.70
Stem diameter (cm)	0.50 ± 0.08	0.62 ± 0.13	0.47 ± 0.15	0.82 ± 0.28	0.70 ± 0.14	0.85 ± 0.28
Q. pubescens						
Stem height (cm)	27.30 ± 11.80	23.80 ± 10.73	32.50 ± 4.95	30.00 ± 2.65	20.33 ± 8.50	20 ± 3.94
Stem diameter (cm)	0.52 ± 0.16	0.66 ± 0.13	0.50 ± 0.14	0.68 ± 0.27	0.58 ± 0.13	0.62 ± 0.04
Q. trojana* subsp. *trojana						
Stem height (cm)	19.50 ± 5.40	28.90 ± 2.88	18.67 ± 4.04	24.20 ± 4.92	18.25 ± 5.30	20.70 ± 1.57
Stem diameter (cm)	0.40 ± 0.14	0.64 ± 0.09	0.38 ± 0.06	0.60 ± 0.07	0.35 ± 0.07	0.56 ± 0.09

Supplementary Material, Table S4: Detailed morphoanatomic features of the ectomycorrhizae (ECM) studied vs. the general features of *T. aestivum* + *Quercus* spp. ECMs [31] according to Agerer & Rambold [30].

	T. aestivum + *Quercus* (Zambonelli et al. 1993)	***Q. coccifera***	***Q. ilex***	***Q. pubescens***	***Q. trojana* subsp. *trojana***	***Q. ithaburnesis* subsp. *macrolepis***
**Mycorrhizal system**						
Ramification type	Absent, or monopodial-pinnate or monopodial-pyramidal	Mainly absent, or monopodial-pinnate or monopodial-pyramidal	Mainly absent, or monopodial-pinnate or monopodial-pyramidal	Mainly absent, or monopodial-pinnate or monopodial-pyramidal	Mainly absent, or monopodial-pinnate or monopodial-pyramidal	Mainly absent, or monopodial-pinnate or monopodial-pyramidal
Abundance		Large clusters of very dense root tips, mainly in the upper part of the root system. The majority of total root tips are distributed in these clusters.	Medium to large clusters of very dense root tips, mainly in the upper part of the root system. The majority of total root tips are distributed in these clusters.	Abundant and very dense solitary root tips, uniformly distributed. Weak to medium root tips clustering, relatively medium in size and number.	Abundant and very dense solitary root tips, uniformly distributed. Weak root tips clustering, relatively small in size and number.	Abundant solitary root tips, uniformly distributed, along with medium clustering of very dense root tips, mainly in the upper part of the root system.
Exploration type	contact	contact	contact	contact	contact	contact
**Unramified ends**						
Mantle hydrophobicity	absent	absent	absent	absent	absent	absent
Shape	straight	straight	straight	straight	straight	straight
Shape of distal ends	not inflated cylindric, or inflated club-shaped	not inflated cylindric, or inflated club-shaped	not inflated cylindric, or inflated club-shaped	not inflated cylindric, or inflated club-shaped	not inflated cylindric, or inflated club-shaped	not inflated cylindric, or inflated club-shaped
Length	(0) 3.2–6.0 mm	0.4 - 0.8(1.2) mm	(0.4) 0.5 - 1.7 (2.1) mm	(0.2) 0.3 - 0.8 (1.1) mm	(0.2) 0.4 - 0.7 (0.8) mm	(0.3) 0.4 - 0.9 (1.1) mm
Diameter	0.3 mm	0.2–0.3 mm / n=30	0.2–0.3 mm / n=30	0.2 - 0.3 mm/ n=25	0.2–0.3 mm/ n=25	0.2 - 0.3 (0.4) mm/ n=25

Color	ochre, yellowish brown	dark brown, brown, brownish	dark brown, brown, brownish	dark brown, brown, brownish	dark brown, brown, brownish	dark brown, brown, brownish
Very tip color	whitish	brownish, sometimes slightly more light-coloured	brownish, sometimes slightly more light-coloured	brownish, sometimes slightly more light-coloured	brownish, sometimes slightly more light-coloured	brownish, sometimes slightly more light-coloured
Older parts color	brown or ochre, yellowish brown	dark- brown	dark- brown	dark- brown	dark- brown	dark- brown
Mantle cortical cells visibility	not visible	not visible	not visible	not visible	not visible	not visible
Mantle surface visibility	visible	visible	visible	visible	visible	visible
Mantle transparency	not transparent	not transparent	not transparent	not transparent	not transparent	not transparent
Mantle dots	-	present at some points, brownish	present at some points, brownish	present at some points, brownish	present at some points, brownish	present at some points, brownish
Mantle surface	densely long-spiny	shiny, or loosely to densely woolly, or densely long-spiny	shiny, or loosely to densely woolly, or densely long-spiny	shiny, or loosely to densely woolly, or densely long-spiny	shiny, or loosely to densely woolly, or densely long-spiny	shiny, or loosely to densely woolly, or densely long-spiny
Emanating hyphae	present, abundant	absent or present according to maturity, abundant when fully mature, concentrated mainly distally towards the tip	absent or present according to maturity, abundant when fully mature, concentrated mainly distally towards the tip	absent or present according to maturity, abundant when fully mature, concentrated mainly distally towards the tip	absent or present according to maturity, abundant when fully mature, concentrated mainly distally towards the tip	absent or present according to maturity, abundant when fully mature, concentrated mainly distally towards the tip
Rhizomorphs	not present	not present	not present	not present	not present	not present
Sclerotia	not present	not present	not present	not present	not present	not present

**Mantle general features**						
Mantle completeness	complete	complete	complete	complete	complete	complete
Mantle organization (in general)		In immature stages, mantle structure starting from plectenchymatous, hyphae with ring-like arrangement (Type A), net-like arrangement repeatedly and squarrosely branched (type E) and/or a net of coarse and irregularly shaped hyphae (type H), gradually changing to pseudoparenchymatous in transition forms of epidermoid cells (type M) to angular cells (type L), and finally pseudoparenchymatous with angular cells (type L) when mature.	In immature stages, mantle structure starting from plectenchymatous, hyphae with ring-like arrangement (Type A), net-like arrangement repeatedly and squarrosely branched (type E) and/or a net of coarse and irregularly shaped hyphae (type H), gradually changing to pseudoparenchymatous in transition forms of epidermoid cells (type M) to angular cells (type L), and finally pseudoparenchymatous with angular cells (type L) when mature.	In immature stages, mantle structure starting from plectenchymatous, hyphae with ring-like arrangement (Type A), net-like arrangement repeatedly and squarrosely branched (type E) and/or a net of coarse and irregularly shaped hyphae (type H), gradually changing to pseudoparenchymatous in transition forms of epidermoid cells (type M) to angular cells (type L), and finally pseudoparenchymatous with angular cells (type L) when mature.	In immature stages, mantle structure starting from plectenchymatous, hyphae with ring-like arrangement (Type A), net-like arrangement repeatedly and squarrosely branched (type E) and/or a net of coarse and irregularly shaped hyphae (type H), gradually changing to pseudoparenchymatous in transition forms of epidermoid cells (type M) to angular cells (type L), and finally pseudoparenchymatous with angular cells (type L) when mature.	In immature stages, mantle structure starting from plectenchymatous, hyphae with ring-like arrangement (Type A), net-like arrangement repeatedly and squarrosely branched (type E) and/or a net of coarse and irregularly shaped hyphae (type H), gradually changing to pseudoparenchymatous in transition forms of epidermoid cells (type M) to angular cells (type L), and finally pseudoparenchymatous with angular cells (type L) when mature.
Emanating elements presence-type	Cystidia	Cystidioid hyphae	Cystidioid hyphae	Cystidioid hyphae	Cystidioid hyphae	Cystidioid hyphae

Emanating elements cystidia location	on outer mantle layer	on outer mantle layer	on outer mantle layer	on outer mantle layer	on outer mantle layer	on outer mantle layer
**Outer mantle layer (apart from tip)**						
Outer mantle organization	pseudoparenchymatous	pseudoparenchymatous	pseudoparenchymatous	pseudoparenchymatous	pseudoparenchymatous	pseudoparenchymatous
Matrix	absent	absent	absent	absent	absent	absent
Mantle type	angular cells (type L)	angular cells (type L)	angular cells (type L)	angular cells (type L)	angular cells (type L)	angular cells (type L)
Cell density (per square, 20 µm x 20 µm)	(6) 7-9.6 (11)	(4) 5-8 (10)	(5) 6-9 (10)	(4) 5-8 (9)	(5) 6-8 (9)	(4) 5-8 (9)
Pores between cells presence	absent	absent	absent	absent	absent	absent
Cell shape	triangular	triangular, quadrilateral or polygonal	triangular, quadrilateral or polygonal	triangular, quadrilateral or polygonal	triangular, quadrilateral or polygonal	triangular, quadrilateral or polygonal
Cell diameter		(3.8) 6.4–12.7 (16) µm / N=30	(6.0) 6.2–10.5 (11.5) µm / N=30	(4.0) 6.7–9.9 (10.1) µm / N=30	(5.3) 7.6–12 (15.1) µm / N=30	(6.4) 7.4–12. 6 (15.4) µm / N=30
Cell walls thickness		0.1 µm	0.1 µm	0.1 µm	0.1 µm	0.1 µm
Cell wall surface habit	-	smooth	smooth	smooth	smooth	smooth
**Emanating elements general features**						
Intrahyphal hyphae	-	not observed	not observed	not observed	not observed	not observed

Backward orientated ramifications	-	not observed	not observed	not observed	not observed	not observed
Anastomoses	-	not observed	not observed	not observed	not observed	not observed
**Emanating hyphae**						
Shape	awl-shaped, bristle-like (type A)	bristle-like, wavy (type A)	bristle-like, wavy (type A)	bristle-like, wavy (type A)	bristle-like, wavy (type A)	bristle-like, wavy (type A)
Pigmentation and contents	-	membranaceously yellowish, without contents	membranaceously yellowish, without contents	membranaceously yellowish, without contents	membranaceously yellowish, without contents	membranaceously yellowish, without contents
Ramifications	-	absent	absent	absent	absent	absent
Septa	simple	simple	simple	simple	simple	simple
Cell shape	-	even	even	even	even	even
Cell diameter	(2.1) 2.6 –3.3 (4.0) µm	(1.4) 1.6 –3.4 (4.5) µm / N=30	(1.7) 2.2 –3.4 (4.0) µm / N=30	(1.7) 2.5 –3.5 (4.2) µm / N=30	(1.7) 2.2 –3.2 (4.0) µm / N=30	(1.7) 2. 6 –4.5 (5.3) µm / N=30
Cell wall surface habit	rough of warts	smooth	smooth	smooth	smooth	smooth
Cell wall thickness		0.1 µm	0.1 µm	0.1 µm	0.1 µm	0.1 µm
Cell wall layers	-	uniform	uniform	uniform	uniform	uniform
Cell wall evenness	-	even thickness	even thickness	even thickness	even thickness	even thickness

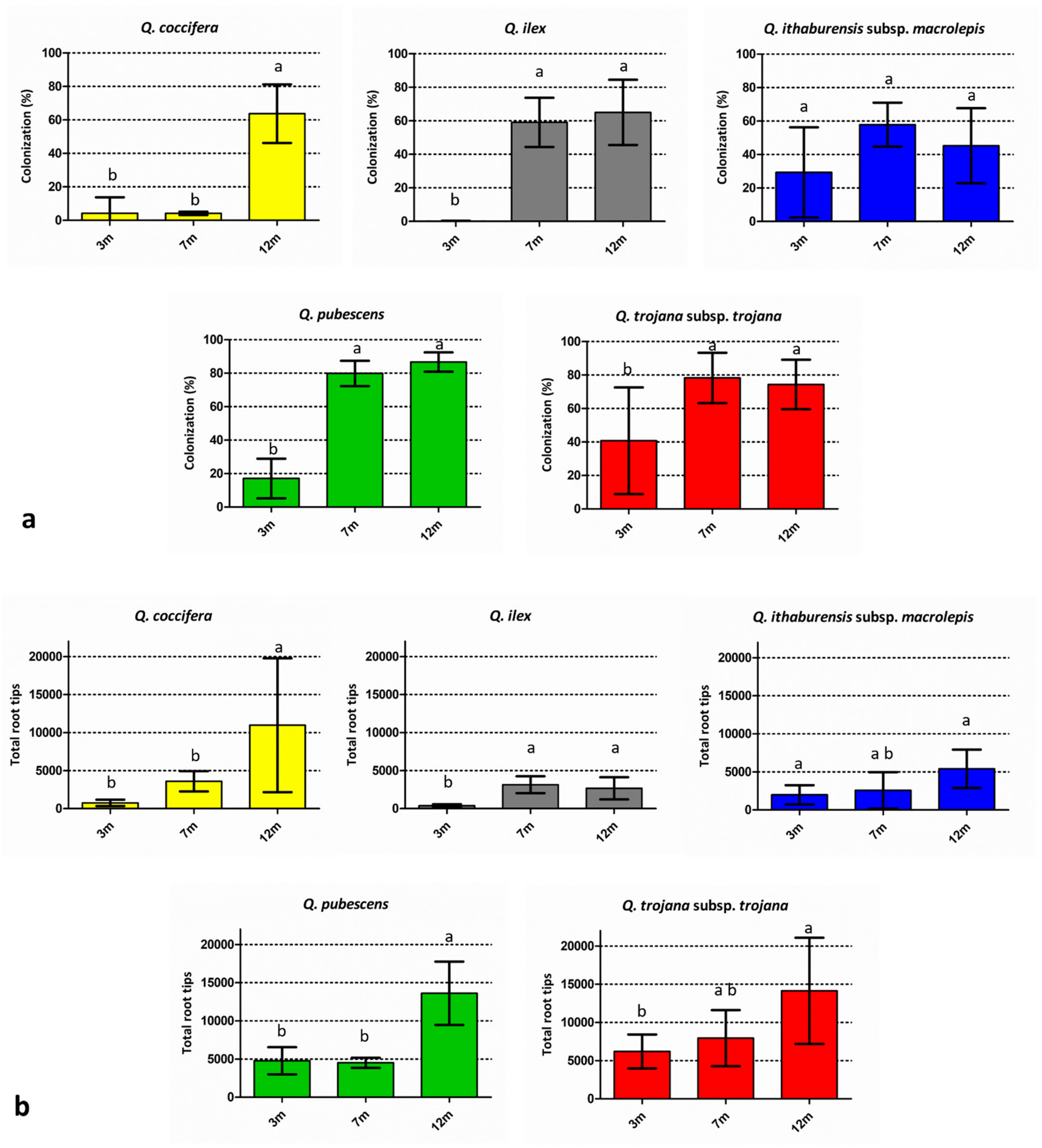

Supplementary Material, Figure S1: (a) mean colonization rates (number of colonized root tips vs. number of total root tips, %), and (b) mean number of total root tips for each *Quercus* species examined. Vertical bars on the columns represent the standard deviation (SD), while absence of common letters indicates significant differences ($p < 0.05$) in comparisons among the evaluation periods (3m, 7m, and 12m: 3-month, 7-month and 12-month, respectively).
